# Use of the Thrombolysis in Myocardial Infarction Risk Index for Elderly Patients With ST-Segment Elevation Myocardial Infarction

**DOI:** 10.3389/fcvm.2021.743678

**Published:** 2021-11-19

**Authors:** Bingqi Fu, Xuebiao Wei, Qi Wang, Zhiwen Yang, Jiyan Chen, Danqing Yu

**Affiliations:** ^1^Division of Cardiology, Guangdong Cardiovascular Institute, Guangdong Provincial Key Laboratory of Coronary Heart Disease Prevention, Guangdong Provincial People's Hospital, Guangdong Academy of Medical Sciences, Guangzhou, China; ^2^Shantou University Medical College, Shantou, China; ^3^Division of Geriatric Intensive Medicine, Guangdong Provincial Geriatrics Institute, Guangdong Provincial People's Hospital, Guangdong Academy of Medical Sciences, Guangzhou, China

**Keywords:** ST-segment elevation myocardial infarction, thrombolysis in myocardial infarction risk index, percutaneous coronary intervention, elderly patients, in-hospital death risk

## Abstract

**Background:** Thrombolysis in Myocardial Infarction (TIMI) Risk Index (TRI) is a simple risk assessment tool for patients with ST-segment elevation myocardial infarction (STEMI). However, its applicability to elderly patients with STEMI undergoing percutaneous coronary intervention (PCI) is uncertain.

**Methods:** This was a retrospective analysis of elderly (≥60 years) patients who underwent PCI for STEMI from January 2010 to April 2016. TRI was calculated on admission using the following formula: heart rate × (age/10)^2^/systolic blood pressure. Discrimination and calibration of TRI for in-hospital events and 1 year mortality were analyzed.

**Results:** Totally 1,054 patients were divided into three groups according to the tertiles of the TRI: <27 (*n* = 348), 27–36 (*n* = 360) and >36 (*n* = 346). The incidence of acute kidney injury (AKI; 7.8 vs. 8.6 vs. 24.0%, *p* < 0.001), AHF (3.5 vs. 6.6 vs. 16.2%, *p* < 0.001), in-hospital death (0.6 vs. 3.3 vs. 11.6%, *p* < 0.001) and MACEs (5.2 vs. 5.8 vs. 15.9%, *p* < 0.001) was significantly higher in the third tertile. TRI showed good discrimination for in-hospital death [area under the curve (AUC) = 0.804, *p* < 0.001; Hosmer-Lemeshow *p* = 0.302], which was superior to its prediction for AKI (AUC = 0.678, *p* < 0.001; Hosmer-Lemeshow *p* = 0.121), and in-hospital MACEs (AUC = 0.669, *p* < 0.001; Hosmer-Lemeshow *p* = 0.077). Receiver-operation characteristics curve showed that TRI > 42.0 had a sensitivity of 64.8% and specificity of 82.2% for predicting in-hospital death. Kaplan-Meier analysis showed that patients with TRI > 42.0 had higher 1 year mortality (Log-rank = 79.2, *p* < 0.001).

**Conclusion:** TRI is suitable for risk stratification in elderly patients with STEMI undergoing PCI, and is thus of continuing value for an aging population.

## Introduction

ST-segment elevation myocardial infarction (STEMI), defined as ST-segment elevation in at least two contiguous leads, has been introduced as a subtype of acute coronary syndrome for purposes of immediate treatment. Percutaneous coronary intervention (PCI) is a class IB treatment in STEMI patients, given that early invasive revascularization therapy can greatly decrease mortality (1). As the elderly population has grown, patients older than 75 years have come to constitute more than 60% of STEMI cases (2). The mean age of candidates for PCI increased by 7 years from 1990 to 2010, and patients aged 75 and over make up 28% of those who undergo PCI in Sweden (3). Age is an independent risk factor for long-term mortality in STEMI, with every one-year increase in age equating to a 1.07 times increase in risk of death (4). The 30-day mortality is 13.4% in 70–79 year-olds and 23.9% in 80 year-olds and above according to the International Survey of Acute Coronary Syndromes in Transitional Countries (ISACS-TC) database (5).

Early risk assessment is necessary for identifying high-risk patients and developing prognoses (1, 2, 4). Thrombolysis in Myocardial Infarction (TIMI) Risk Index (TRI) was created by Morrow et al. (6) to guide rapid initial triage for STEMI patients. It has performed well in predicting 30 day mortality in the general population of patients who underwent revascularization therapy; however, the proportion of patients who received PCI was small, being <3% in Bradshaw et al. (7) 4.4% in Wiviott et al. (8) and 5.4% in Rathore et al. (9) The value of the TRI has not been confirmed in the context of PCI being the mainstay of therapy for STEMI. In an attempt to provide first-line information regarding Chinese patients, we evaluated the use of TRI to predict mortality and clinical events among elderly STEMI patients who received PCI.

## Methods

### Study Design and Population

**Figure 1:**
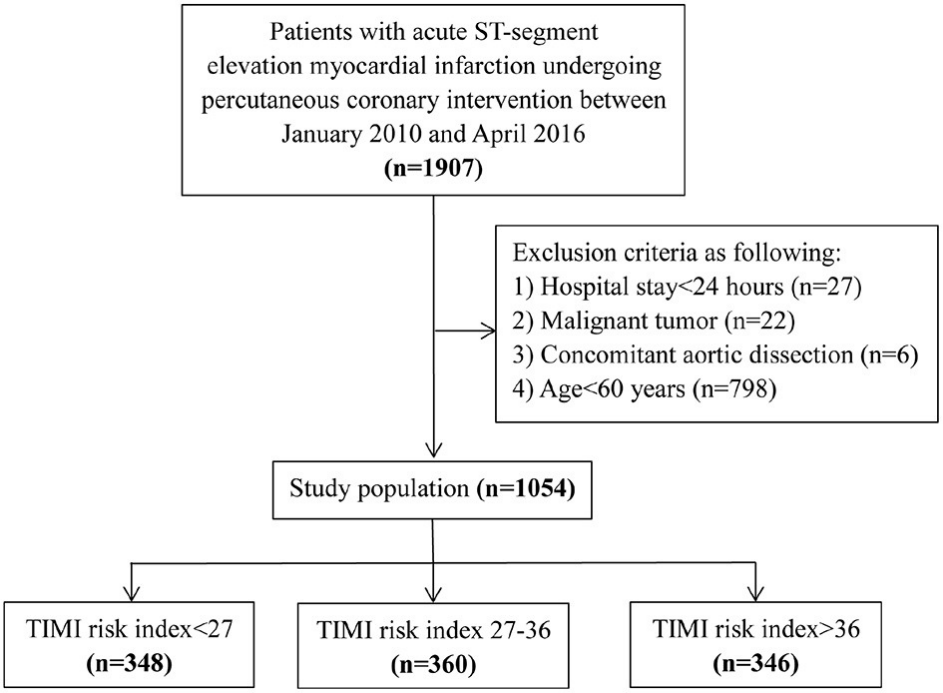
Flow diagram for patient screening.

Ours was a retrospective study approved by the Ethics Committee of Guangdong Provincial People's Hospital with a waiver of informed consent because of the retrospective study design (no. GDREC2016411H). Statistical analysis was performed on the patient population and identifying information was strictly concealed during the study. We consecutively enrolled 1907 patients with STEMI who had undergone PCI at cardiac care unit (CCU) from January 2010 to April 2016 at Guangdong Provincial People's Hospital, Guangzhou, China. The definition of STEMI was taken from the American College of Cardiology Foundation/American Heart Association Task Force (10). After excluding patients with a hospital stay of <24 h (*n* = 27), a malignant tumor (*n* = 22), concomitant aortic dissection (*n* = 6) or age <60 years (*n* = 798), 1,054 patients were included in our study population (Figure 1).

### Data Extraction

Data was extracted from hospital records by trained study coordinators. Data regarding patient demographics [i.e., age, gender, weight, heart rate, systolic blood pressure (SBP), diastolic blood pressure (DBP), diabetes mellitus, hypertension, time to admission, anatomical location of myocardial infarction, Killip classification, hospital stay], laboratory test results [i.e., serum creatinine, hemoglobin, creatine kinase myocardial band (MB)], ultrasonographic results, [i.e., left ventricular ejection fraction (LVEF)], medical treatment, PCI details [i.e., use of intra-aortic balloon pump (IABP), thrombus aspiration, vessels treated, number of stents], major adverse clinical events (MACEs), acute kidney injury (AKI) and in-hospital death were collected. All in-hospital clinical events were recorded by two independent researchers who were not informed of the treatments. Other data was collected by one researcher and randomly checked by another researcher. The TRI was calculated on admission using the following formula: heart rate × (age/10)^2^/systolic blood pressure (6).

### Study Endpoints

All patients were monitored for 1 year by trained nurses via telephone interviews or clinical visits. Oral informed consents were obtained during the first telephone interview or the first clinical visit after discharge. The data of death was recorded during follow-up and 1 year death was determined according to the date of death. The primary endpoint of the study was in-hospital death. The secondary endpoint was in-hospital MACEs, AKI, acute heart failure (AHF) or death during 1 year of follow-up. In-hospital MACEs were a composite end point of renal dialysis, stroke, target vessel revascularization (TVR). AKI was defined as a post-PCI rise in serum creatinine (sCr) ≥ 0.3 mg/dl, or a ≥ 50% elevation from baseline over the course of hospitalization as per the Kidney Disease International Global Outcomes Guidelines (11). Acute heart failure was defined as newly onset or worsening of symptoms and signs of pre-existing heart failure that required intravenous therapy (inotropes, vasodilators, or diuretics) (12, 13).

### Statistical Analysis

Normally distributed continuous variables were shown as mean ± standard deviation, while those with a skewed distribution were shown as median with upper and lower quartiles. Categorial variables were shown as percentages. Continuous variables were compared using variances or the Wilcoxon rank-sum test, while categorial variables were compared using the chi-square test. The cut-off value was calculated using the receiver-operation characteristics (ROC) curve analysis. All variables were included in the univariate models to assess the predictive value of TRI for adverse events. Variables with a *p* < 0.05 were then incorporated in the multivariate models for further evaluation. Use of the TRI in predicting adverse events was evaluated by analysis of discrimination and calibration. Discriminative performance was expressed as the area under curve (AUC) of ROC curve. Calibration was expressed as the Hosmer-Lemeshow (H-L) chi-square. For 1 year cumulative survival analysis, Kaplan-Meier curves were acquired and log-rank tests were performed. All data was processed using SPSS software version 22.0 (SPSS, Inc., Chicago, Illinois). A two-sided *p* < 0.05 was considered statistically significant.

## Results

### Baseline Characteristics

A total of 1,054 patients (mean age: 70 ± 7 years; gender: 25.5% female) were divided into three groups according to the TRI tertiles: <27 (*n* = 348), 27–36 (*n* = 360) and >36 (*n* = 346). Comparisons among the three groups revealed significant differences between the following variables: age, weight, anterior myocardial infarction, systolic blood pressure, diastolic blood pressure, heart rate, Killip II-IV, serum creatinine, hemoglobin, LVEF, use of IABP and length of hospital stay (all *p* < 0.05, Table 1).

**Table 1:** Clinical characteristics for TRI tertiles.

**Variable**	**TIMI Risk Index (TRI)**			***P*** **-value**
	**<27 (***n*** = 348)**	**27–36 (***n*** = 360)**	**>36 (***n*** = 346)**	
Age, years	65.4 ± 4.9	70.3 ± 6.1	75.0 ± 6.4	<0.001
Female sex, n (%)	75 (21.6)	96 (26.7)	98 (28.3)	0.102
Diabetes mellitus, n (%)	88 (25.3)	103 (28.6)	99 (28.6)	0.524
Hypertension, n (%)	223 (64.1)	210 (58.3)	206 (59.5)	0.259
Weight, kg	64.4 ± 10.3	62.0 ± 9.7	61.0 ± 10.2	<0.001
Time to admission <6 h, *n* (%)	92 (26.4)	92 (25.6)	89 (25.7)	0.961
Anterior myocardial infarction, *n* (%)	141 (40.5)	157 (43.6)	184 (53.2)	0.002
SBP, mmHg	134.4 ± 22.2	122.9 ± 19.2	110.7 ± 19.8	<0.001
DBP, mmHg	77.0 ± 13.2	71.9 ± 11.6	68.0 ± 12.6	<0.001
Heart rate, bpm	70.7 ± 12.8	77.9 ± 12.9	89.6 ± 15.4	<0.001
Killip II-IV, *n* (%)	85 (24.4)	115 (31.9)	176 (50.9)	<0.001
Serum creatinine, mg/dL	84.0 (70.4,100.0)	88.0 (72.1,109.0)	100.5 (79.1,133.2)	<0.001
Hemoglobin, g/L	130.6 ± 15.7	128.2 ± 15.1	124.1 ± 18.0	<0.001
Creatine kinase MB, U/L	61.7 (25.2,128.1)	57.4 (24.6,142.9)	66.2 (24.4,148.8)	0.360
LVEF, %	55.0 ± 9.7	52.0 ± 11.0	47.9 ± 12.4	<0.001
IABP, *n* (%)	20 (5.7)	33 (9.2)	91 (26.3)	<0.001
Thrombus aspiration, *n* (%)	109 (31.3)	119 (33.1)	109 (31.5)	0.862
**Treated vessel**,***n*** **(%)**				
Any left main	15 (4.3)	16 (4.4)	23 (6.6)	0.604
Multi-vessel	40 (11.5)	38 (10.6)	35 (10.1)	
Others	293 (84.2)	306 (85.0)	288 (83.2)	
Number of stents ≥ 3, *n* (%)	31 (8.9)	24 (6.7)	35 (10.1)	0.249
Hospital stay (days)	6 (5,8)	7 (6,9)	9 (6,14)	<0.001
**In-hospital events**				
AKI	27 (7.8)	31 (8.6)	83 (24.0)	<0.001
AHF*	12 (3.5)	23 (6.6)	50 (16.2)	<0.001
Death	2 (0.6)	12 (3.3)	40 (11.6)	<0.001
MACEs	18 (5.2)	21 (5.8)	55 (15.9)	<0.001

*SBP, systolic blood pressure; DBP, diastolic blood pressure; LVEF, left ventricular ejection fraction; IABP, intra-aortic balloon pump; AKI, acute kidney injury; AHF, acute heart failure; MACEs, major adverse clinical events*.

* *Patients with Killip IV on admission were not included*.

### Clinical Outcomes

The overall incidence of in-hospital death, AKI, AHF and MACEs was 54 (5.1%), 141 (13.4%), 85(8.5%) and 94(8.9%), respectively (Table 2). The frequency of clinical events varied significantly among the different groups. The incidence of AKI (7.8 vs. 8.6 vs. 24.0%, *p* < 0.001), AHF (3.5 vs. 6.6 vs. 16.2%, *p* < 0.001), in-hospital death (0.6 vs. 3.3 vs. 11.6%, *p* < 0.001) and MACEs (5.2 vs. 5.8 vs. 15.9%, *p* < 0.001) was significantly higher in the third tertile (Table 1).

**Table 2:** Validation of TRI score.

**Events**	**Incidence**	**AUC (95%CI)**	**H-L**	**H-L** ***p*****-value**
In-hospital death	54 (5.1)	0.804 (0.745–0.863)	9.5	0.302
AKI	141 (13.4)	0.678 (0.628–0.728)	12.7	0.121
AHF*	85 (8.5)	0.705 (0.645–0.764)	6.9	0.546
MACEs	94 (8.9)	0.666 (0.600–0.733)	14.2	0.077

*AUC, area under the curve; H-L, Hosmer-Lemeshow; AKI, acute kidney injury; AHF, acute heart failure; MACEs, major adverse clinical events*.

* *Patients with Killip IV on admission were not included*.

**Figure 2:**
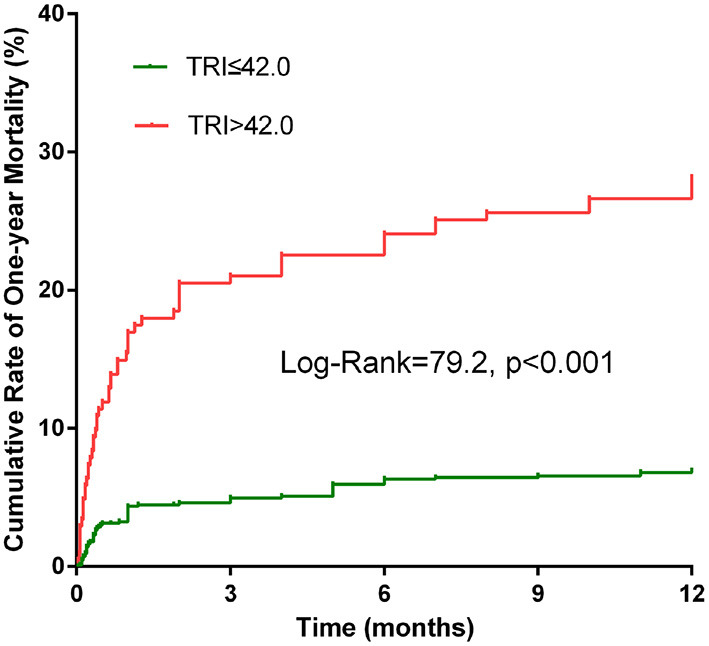
Kaplan-Meier analyses for 1 year mortality according to the TRI categories.

Univariate analysis showed a significant correlation between TRI and AKI, in-hospital death, in-hospital MACEs and 1 year mortality. After adjustment for potential confounding factors, TRI remained to be significantly associated with AKI (OR = 1.02, 95% CI 1.01–1.04, *p* = 0.007), AHF (OR = 1.03, 95% CI 1.01–1.05, *p* = 0.005) in-hospital death (OR = 1.05, 95%CI 1.03–1.07, *p* < 0.001), in-hospital MACEs (OR = 1.03, 95%CI 1.01–1.05, *p* = 0.006) and 1 year mortality (HR = 1.03, 95% CI 1.02–1.04, *p* < 0.001, Table 3). ROC curve revealed that 42.0 was the optimal cut-off value of the TRI for predicting in-hospital death, with the sensitivity and specificity being 64.8% and 82.2%, respectively. The Kaplan-Meier curves showed that patients with TRI > 42.0 had higher cumulative 1 year mortality than those with TRI ≤ 42.0 (Log-rank = 79.2, *p* < 0.001, Figure 2). Multivariate analysis revealed that TRI > 42.0 was an independent risk factor for AKI (OR = 1.86, 95% CI 1.20–2.88, *p* = 0.006), AHF (OR = 1.77, 95% CI 1.03–3.04, *p* = 0.040), in-hospital death (OR = 3.76, 95% CI 1.97–7.18, *p* < 0.001), in-hospital MACEs (OR = 1.75, 95% CI 1.02–3.02, *p* = 0.044), and 1 year mortality (HR = 2.23, 95% CI 1.47–3.36, *p* < 0.001, Table 3).

**Table 3:** Unadjusted and adjusted OR/HR of TRI for adverse events.

	**TRI**		**TRI > 42.0 vs. TRI ≤ 42.0**	
	**OR/HR (95%CI)**	***P*** **-value**	**OR/HR (95%CI)**	***P*** **-value**
**AKI**				
Model 1: unadjusted	1.03 (1.04,1.06)	<0.001	3.38 (2.31,4.94)	<0.001
Model 2: multivariate adjusted^a^	1.02 (1.01,1.04)	0.007	1.86 (1.20,2.88)	0.006
**In-hospital AHF**				
Model 1: unadjusted	1.05 (1.04,1.07)	<0.001	3.49 (2.18,5.60)	<0.001
Model 2: multivariate adjusted^b^	1.03 (1.01,1.05)	0.005	1.77 (1.03,3.04)	0.040
**In-hospital death**				
Model 1: unadjusted	1.07 (1.05,1.10)	<0.001	7.57 (4.27,13.39)	<0.001
Model 2: multivariate adjusted^c^	1.05 (1.03,1.07)	<0.001	3.76 (1.97,7.18)	<0.001
**In-hospital MACEs**				
Model 1: unadjusted	1.05 (1.03,1.06)	<0.001	4.15 (2.68,6.45)	<0.001
Model 2: multivariate adjusted^d^	1.03 (1.01,1.05)	0.006	1.75 (1.02,3.02)	0.044
**1 year mortality**				
Model 1: unadjusted	1.04 (1.03,1.05)	<0.001	4.58 (3.17,6.62)	<0.001
Model 2: multivariate adjusted^e^	1.03 (1.02,1.04)	<0.001	2.23 (1.47,3.36)	<0.001

*a, Adjusted for diabetes mellitus, hypertension, Killip II-IV, eGFR <60ml/min/1.73m^2^, anemia, lgCKMB, LVEF and IABP. b, Adjusted for diabetes mellitus, Killip II-IV, eGFR <60ml/min/1.73m^2^, LVEF and IABP. c, Adjusted for Killip II-IV, eGFR <60 ml/min/1.73 m^2^, lgCKMB, LVEF and IABP. d, Adjusted for diabetes mellitus, Killip II-IV, eGFR <60 ml/min/1.73 m^2^, anemia, LVEF and IABP. e, Adjusted for diabetes mellitus, time to admission <4 h, Killip II-IV, eGFR <60 ml/min/1.73 m^2^, LVEF and IABP. AKI, acute kidney injury; MACEs, major adverse clinical events; OR, odds ratio; HR, hazard ratio; CI, confidence interval; eGFR, effective glomerular filtration rate; lgCKMB, the common logarithm of creatine kinase-MB; LVEF, left ventricular ejection fraction; IABP, intra-aortic balloon pump*.

### Validation of the TRI

**Figure 3:**
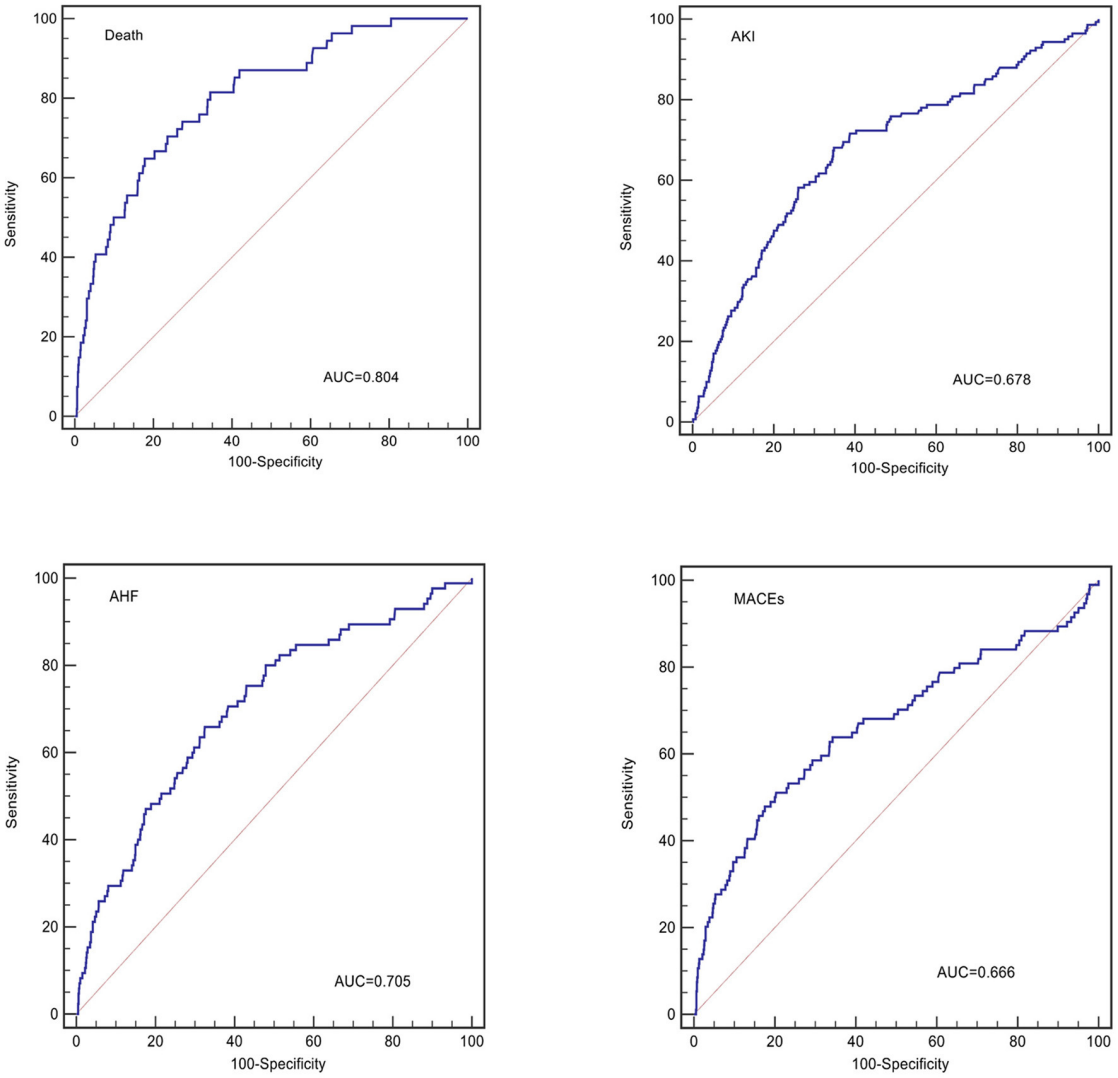
The receiver-operation characteristics curve of the TRI for predicting in-hospital events.

**Figure 4:**
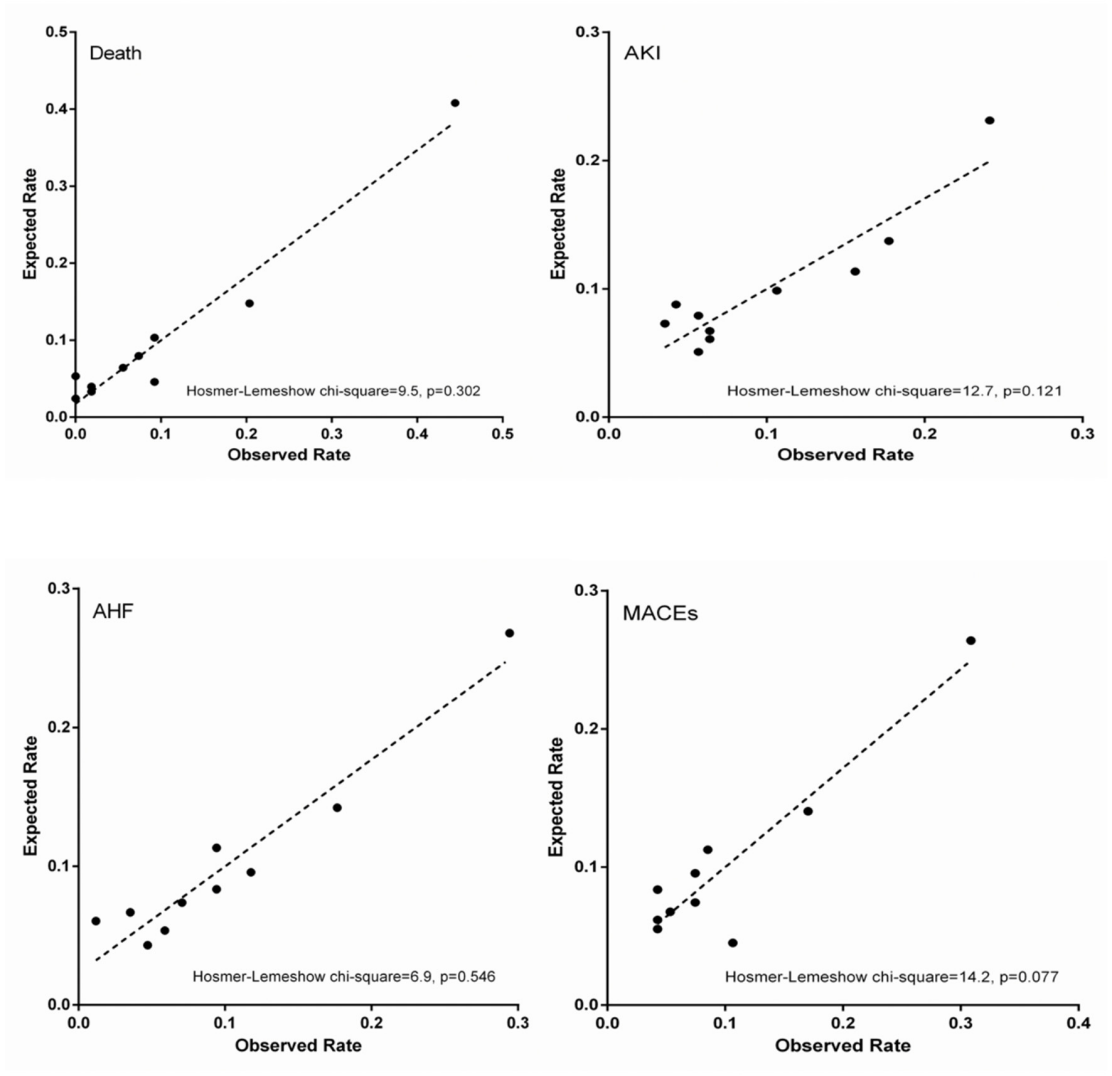
Calibration plots.

The TRI showed good discrimination for in-hospital death (AUC = 0.804, 95% CI 0.779–0.828, *p* < 0.001, Table 2, Figure 3), but relatively poor discrimination for AKI (AUC = 0.678, 95% CI 0.649–0.706, *p* < 0.001, Table 2, Figure 3), AHF (AUC = 0.705, 95% CI 0.645–0.764, *p* < 0.001, Table 2, Figure 3), and in-hospital MACEs (AUC = 0.669, 95% CI 0.640–0.698, *p* < 0.001, Table 2, Figure 3). As for calibration, there was no significant difference between expected and observed events when the TRI was used to predict in-hospital death (H-L chi-square = 9.5, *p* = 0.302, Table 2, Figure 4), AKI (H-L chi-square = 12.7, *p* = 0.121, Table 2, Figure 4), AHF (H-L chi-square = 6.9, *p* = 0.546, Table 2, Figure 4), and in-hospital MACEs (H-L chi-square = 14.2, *p* = 0.077, Table 2, Figure 4). Overall, the TRI had good discrimination for in-hospital death, which was superior to its prediction for AKI, AHF and in-hospital MACEs.

### Subgroup Analysis

According to the anatomical location of myocardial infarction, patients were subdivided into anterior myocardial infarction subgroup (TRI = 34.8 ± 11.8) and non-anterior myocardial infarction subgroup (TRI = 32.2 ± 12.0). The predictive value TRI for in-hospital death was slightly higher in anterior myocardial infarction subgroup (AUC = 0.837, 95% CI 0.774–0.899, H-L *p* = 0.151) than that in non-anterior myocardial infarction (AUC = 0.776, 95% CI 0.682–0.869, H-L *p* = 0.205, Table 4).

**Table 4:** Subgroup analysis for predicting in-hospital death.

**Subgroup**	**TRI**	**AUC (95%CI)**	**H-L *P*-value**
Anterior myocardial infarction	34.8 ± 11.8	0.837 (0.774,0.899)	0.151
Non-anterior myocardial infarction	32.2 ± 12.0	0.776 (0.682,0.869)	0.205

*AUC, area under the curve; CI, confidence interval; H-L, Hosmer–Lemeshow*.

## Discussion

To our best knowledge, this study is the first to validate the efficacy of the TRI in elderly patients receiving PCI. The results show that the TRI has good discrimination and calibration for in-hospital death, but not for AKI or in-hospital MACEs. Therefore, the TRI is suitable for predicting in-hospital mortality in elderly patients with STEMI who have undergone PCI.

PCI has gradually become a mainstream therapy of STEMI that has benefited survival. The most recent epidemiological study in Europe reported a decline in STEMI 30 day mortality over two decades, from 14% in 1995 to 3% in 2015 (14). In terms of the elderly, however, the prognosis remained poor (3–5), and early identification of high-risk patients was essential in clinical practice (1, 2, 4). The TRI was initially derived from the InTIME II trial by Morrow et al. (6) which involved 13,253 STEMI patients undergoing thrombolytic therapy with an in-hospital mortality of 5.4% and a 30 day mortality of 6.0%. It demonstrated good discrimination (AUC = 0.78) and calibration (Pearson goodness-of-fit statistic = 2.83, *p* = 0.7) for 30 day mortality among STEMI patients with thrombolysis. Rathore et al. (9) analyzed patients ≥ 65 years of age from the Cooperative Cardiovascular Project (CCP) database and showed that the discrimination (AUC = 0.62) and calibration (goodness of fit *p* < 0.001) of TRI for predicting 30 day mortality were unsatisfying, while Bradshaw et al. (AUC = 0.74) and Wiviott et al. (AUC = 0.71) (7, 8) showed slightly higher predictive value. These studies primarily evaluated patients who received thrombolytic or fibrinolytic therapy, while primary PCI played only a small part. The proportion of PCI was <3% in Bradshaw et al. (7) 4.4% in Wiviott et al. (8) and 5.4% in Rathore et al. (9). Thus, clinical application of the TRI in elderly STEMI patients who received PCI had yet to be tested. In our study we demonstrated good discriminative capacity with AUC of 0.804 and good calibration of the TRI in predicting 30 day mortality in elderly STEMI patients who received PCI. Given that the TRI is formulated using easily obtained variables, i.e., age, heart rate and blood pressure, it is promising for rapid risk stratification in clinical practice.

Clinical adverse events such as congestive heart failure, in-hospital death, and acute kidney injury following STEMI are known to frequently be correlated with a higher TRI, but the predictive value of the TRI for these potential clinical adverse events was unknown (15–18). In our study, the ability of the TRI to predict AKI and in-hospital MACEs were relatively poor. This was probably the result of inadequate involvement of independent risk factors (19, 20). Age is an independent risk factor for AKI and in-hospital MACEs (16, 17, 21), and since age is one component of the TRI, this might explain why the TRI was partially, though poorly, predictive of these events. Apart from age, there are many other variables that contribute to specific clinical events. Variables such as serum creatinine, estimated glomerular filtration rate, contrast media volume, and underlying comorbidities (i.e., hypertension, diabetes mellitus, etc.) anticipate the rate of AKI and the need for renal dialysis (11, 16, 22), but are not included in the TRI. Nor are stroke-associated predictors such as carotid artery disease and atrial fibrillation (23). As for repeat coronary revascularization, Atti et al. (24) showed in a recent meta-analysis that risk of multivessel revascularization was reduced by 66% compared to culprit-only revascularization, which is also not incorporated into the TRI. Since most variables that were valuable for predicting the above-mentioned clinical events are only obtained from laboratory results and PCI, risk assessment is delayed. The TRI, on the other hand, can provide rapid initial triage of STEMI patients.

The discriminative capacity and calibration of TRI for predicting mortality in elderly STEMI patients in our study was better than that in Rathore's study, which might be explained by the variation of baseline characteristics and mortality. Compared to CCP-RT cohort, our study cohort varied in terms of patient demographics, medical history, admission characteristics and short-term mortality (Table 5) (9). On one hand, our study cohort included Asian ethnic people, while CCP-RT cohort recruited majorly white ethnic people. Other variables including proportion of female patients, medical history of myocardial infarction, on-admission status such as shock, systolic pressure and timing on arrival, were all possibly responsible for the different discriminative and calibration results of TRI between CCP-RT cohort and our cohort. On the other hand, our cohort had a 9.5% reduction of short-term mortality compared to CCP-RT cohort, which might be due to the fact that only 5.4% of CCP-RT cohort received PCI therapy, while the rest received thrombolytic therapy (9). PCI therapy has now recognized as the preferred reperfusion therapy within 120 min of STEMI diagnosis, as it benefits the clinical outcomes compared to thrombolysis (25).

**Table 5:** Comparison of patient characteristics.

**Variable**	**CCP-RT cohort (*n* = 18,089)**	**Our cohort (*n* = 1,054)**
**Demographics**		
Age, years (IQR)	73 (69–78)	70 (64–76)
Female sex (%)	43.8	25.5
White (%)	92.2	0.0
**Medical history (%)**		
Prior MI	20.2	5.7
Peripheral vascular disease	6.5	2.4
Cerebrovascular disease	7.4	9.2
Diabetes mellitus	23.7	27.5
Hypertension	56.1	60.6
Current smoking	20.7	33.6
**Admission characteristics**		
Shock (%)	2.6	8.3
Anterior MI or LBBB (%)	59.0	45.7
Pulse, bpm (IQR)	77 (65–90)	78 (68–90)
SBP, mmHg (IQR)	140 (120–160)	121(107–136)
Arrived within 6 h (%)	80.4	25.9
**Short-term mortality (%)**	14.6	5.1

*CCP, Cardiovascular Cooperative Project; RT, reperfusion therapy; IQR, interquartile range; MI, myocardial infarction; LBBB, left bundle-branch block; SBP, systolic blood pressure*.

## Limitation

Our study had several limitations. Firstly, although the TRI has the advantage of rapid risk assessment, caution should be applied to long-term prognosis, which is a combination of multiple intertwined factors such as acute physiological change (e.g., serum creatinine, white blood cell count, etc.), timing of PCI, location of culprit vessels, in-hospital complications, frailty and cognitive function (9, 20, 26–30). Therefore, after initial triage, risk assessment should be updated dynamically through treatment to more accurately predict mortality (1, 8). Secondly, our study was designed as a retrospective analysis, and although bias was therefore unavoidable, efforts were made to minimize it. Finally, the study population was relatively small, and the results might be different with a larger cohort. Multi-centered, prospective studies with larger samples are needed to confirm our results.

## Conclusion

In summary, the TRI remains suitable for risk stratification in elderly patients with STEMI who underwent PCI. The TRI has advantages of good discrimination and calibration, as well as a simple formula that allows rapid initial risk assessment.

## Data Availability Statement

The raw data supporting the conclusions of this article will be made available by the authors, without undue reservation.

## Ethics Statement

The studies involving human participants were reviewed and approved by the Ethics Committee of Guangdong Provincial People's Hospital. Written informed consent for participation was not required for this study in accordance with the national legislation and the institutional requirements.

## Author Contributions

DY and JC were involved in the conception and design of this study. BF, XW, ZY, and QW contributed to data collection and data interpretation. BF, XW, and QW constructed the manuscript, which was revised and approved by all the authors for publication.

## Funding

This study was supported by grants from National Natural Science Foundation of China (Grant No. 82002014), Natural Science Foundation of Guangdong Province (Grant No. 2021A1515010107), Science and Technology Projects of Guangzhou (Grant No. 201903010097), and Guangdong Provincial Key Laboratory of Coronary Heart Disease Prevention (Grant No. 2017B030314041). The funders had no role in the study design, data collection and analysis, decision to publish, nor preparation of the manuscript.

## Conflict of Interest

The authors declare that the research was conducted in the absence of any commercial or financial relationships that could be construed as a potential conflict of interest.

## Publisher's Note

All claims expressed in this article are solely those of the authors and do not necessarily represent those of their affiliated organizations, or those of the publisher, the editors and the reviewers. Any product that may be evaluated in this article, or claim that may be made by its manufacturer, is not guaranteed or endorsed by the publisher.
